# A Novel Simple Method for Determining *CYP2D6* Gene Copy Number and Identifying Allele(s) with Duplication/Multiplication

**DOI:** 10.1371/journal.pone.0113808

**Published:** 2015-01-27

**Authors:** Taimour Langaee, Issam Hamadeh, Arlene B. Chapman, John G. Gums, Julie A. Johnson

**Affiliations:** 1 Department of Pharmacotherapy and Translational Research and Center for Pharmacogenomics, College of Pharmacy, University of Florida, Gainesville, Florida, United States of America; 2 Renal Division, Department of Medicine, Emory University School of Medicine, Atlanta, Georgia, United States of America; Odense University hospital, DENMARK

## Abstract

**Background:**

Cytochrome P450 2D6 (*CYP2D6*) gene duplication and multiplication can result in ultrarapid drug metabolism and therapeutic failure or excessive response in patients. Long range polymerase chain reaction (PCR), restriction fragment length polymorphism (RFLP) and sequencing are usually used for genotyping *CYP2D6* duplication/multiplications and identification, but are labor intensive, time consuming, and costly.

**Methods:**

We developed a simple allele quantification-based Pyrosequencing genotyping method that facilitates *CYP2D6* copy number variation (CNV) genotyping while also identifying allele-specific *CYP2D6* CNV in heterozygous samples. Most routine assays do not identify the allele containing a CNV. A total of 237 clinical and Coriell DNA samples with different known *CYP2D6* gene copy numbers were genotyped for *CYP2D6* *2, *3, *4, *6, *10, *17, *41 polymorphisms and CNV determination.

**Results:**

The *CYP2D6* gene allele quantification/identification were determined simultaneously with CYP2D6*2, *3, *4, *6, *10, *17, *41 genotyping. We determined the exact *CYP2D6* gene copy number, identified which allele had the duplication or multiplication, and assigned the correct phenotype and activity score for all samples.

**Conclusions:**

Our method can efficiently identify the duplicated *CYP2D6* allele in heterozygous samples, determine its copy number in a fraction of time compared to conventional methods and prevent incorrect ultrarapid phenotype calls. It also greatly reduces the cost, effort and time associated with *CYP2D6* CNV genotyping.

## Introduction

The cytochrome CYP2D6 is responsible for the metabolism of more than 30% of all orally administered drugs including many antipsychotics, antidepressants, antiarrhythmics, and opioid analgesics [1]. The interest in studying the *CYP2D6* gene continues to grow because of its significant contribution to interindividual variability in drug metabolism, resulting in higher incidence of adverse events or lack of therapeutic efficacy.

The *CYP2D6* gene is located on chromosome 22, and is flanked by two pseudogenes *CYP2D7* and *CYP2D8* that share 95% sequence homology with *CYP2D6* [2, 3]. Owing to its highly polymorphic nature, more than 100 *CYP2D6* variant alleles have been identified to date (www.cypalleles.ki.se). The genetic variations in *CYP2D6* results in four different drug metabolism phenotypes; poor metabolizer (PM), intermediate metabolizer (IM), extensive metabolizer (EM), and ultrarapid metabolizer (UM). The latter is the result of gene duplication/multiplication and occurs with inheritance of more than two copies of the fully functional *CYP2D6* alleles [4].

Current genotyping techniques targeting the *CYP2D6* variant alleles have facilitated phenotype prediction from the genotype information without the need for the administration of CYP2D6 probe drugs [5]. Hence, the correct genotyping and genotype to phenotype translation is very important in a clinical setting where this information may serve as a guide for individualization of drug therapy [4].

The presence of different genetic variations in *CYP2D6* such as single nucleotide polymorphisms (SNPs), short insertion and deletions, gene conversions, copy number variations (CNVs), including gene deletion and duplication/multiplications of whole gene, mandates the use of more than one assay to determine the *CYP2D6* genotypes [3, 6]. Additionally, in certain cases of gene duplications or multiplications, the process of deriving phenotypes from genotypes via the activity score system becomes difficult because most of the copy number assays do not identify which of the two alleles carries the duplication or multiplication. Long range PCR, sequencing or Southern-RFLP methods are typically used to resolve this issue [3, 7, 8, 9, 10, 11]. However, these methods are laborious and costly and hence impractical in clinical settings where rapid retrieval of phenotype information is often warranted.

In this study we describe a simple rapid allele quantification-based method by pyrosequencing that can identify the duplicated *CYP2D6* allele and estimate its copy number in heterozygous samples that carry duplications/multiplications of the *CYP2D6* gene.

## Materials and Methods

### DNA samples

A total of 218 DNA samples from the Pharmacogenomic Evaluation of Antihypertensive Responses (PEAR-II) clinical study (clinical trial.gov identifier: NCT01203852) that were isolated from blood leukocytes by the FlexiGene DNA Kit (Qiagen, Valencia, CA, USA) were used to genotype *CYP2D6* SNPs and copy number variations. All the subjects from the PEAR-II clinical study (clinical trial.gov identifier: NCT01203852) provided written informed consent to participate for screening before active enrollment and supply genetic material. The study protocol was approved by the institutional review board of each participating institution [12]. These 218 samples were collected from 125 Caucasians (57.5%), 84 African Americans (38.5%), 1 Asian (0.5%), and 8 other (3.5%). Eighteen (8.25%) of 218 PEAR-II DNA samples with different *CYP2D6* gene copy numbers (1, 2, 3, and 4 copies) and all those with duplications or multiplications were selected for further analysis in this study [12]. These 18 samples along with another 19 DNA samples (Table 1) [13] with known *CYP2D6* copy number purchased from Coriell Cell Repository (http://ccr.coriell.org/) were used in this study to evaluate the new *CYP2D6* pyrosequencing allele quantification-based genotyping method. The Coriell DNA samples with their genotypes are shown in Table 1.

**Table 1:** Coriell DNA samples used for validating *CYP2D6* allele quantification assay.

**ID #**	**Genotype**
NA18564	*1/*10×2
NA18532	(*10/*10)×4
NA18545	(*10/*10)×4
NA18944	(*10/*10)×4
NA18960	(*10/*10)×4
NA18603	(*10/*10)×4
NA18561	(*10/*10)×4
NA18959	*2/*10×2
NA18537	*1/*10×2
NA18542	*1/*10×2
NA18524	*1/*10×3
NA18526	*1/*10×3
NA18563	*1/*10×2
NA18947	*1/*10×2
GM17221	*1x2/*2
GM02016	*2x2/*17
GM17298	(*1/*1)xN
GM17232	(*2/*2)xN
GM17244	*2/*4

### Pyrosequencing assay design

The pyrosequencing assays for CYP2D6*2 (2850 C>T, rs16947), *3 (2549 del A, rs35742686), *4 (1846 G>A, rs3892097), *6 (1707 del T, rs5030655), *10 (100 C>T, rs1065852), *17 (1023 C/T, rs28371706), and *41 (2988 G>A, rs28371725) alleles were separately designed (Table 2). The PCR and sequencing primers for each assay were designed by Pyrosequencing Primer Design Software (Qiagen, Valencia, CA, USA) and are shown in Table 2.

**Table 2:** Pyrosequencing PCR and sequencing primers for CYP2D6*2, *3, *4, *6*10, *17, *41 alleles.

**CYP2D6 alleles**	**PCR Primer**	**Pyrosequencing Primer**
*2 (2850 C>T) rs16947	F. Bio-GGCCCCTGCACTGTTTCC	R-CAGCTTCAATGATGAGAAC
*2 (2850 C>T) rs16947	R-AAGGGGAACCCTGAGAGC	R-CAGCTTCAATGATGAGAAC
*3 (2549 del A) rs35742686	F-CTGTCCCCGTCCTCCTGCAT	F-AGCTGCTAACTGAG
*3 (2549 del A) rs35742686	R. Bio-CCTCATTCCTCCTGGGACGC	F-AGCTGCTAACTGAG
*4 (1846 G>A) rs3892097	F-TGCCGCCTTCGCCAACCACT	F-CCGCATCTCCCACCCC
*4 (1846 G>A) rs3892097	R. Bio-GCAGAGACTCCTCGGTCTCTC	F-CCGCATCTCCCACCCC
*6 (1707 del T) rs5030655	F-CTAATGCCTTCATGGCCACGCG	F-GAAGTCGCTGGAGCA
*6 (1707 del T) rs5030655	R. Bio-GCTTTGTGCCCTTCTCCCATCA	F-GAAGTCGCTGGAGCA
*10 (100 C>T rs1065852	F.Bio- TGTCCAGAGGAGCCCATTT	R- GGCAGGGGGCCTGGT
*10 (100 C>T rs1065852	R- GTCGAAGCAGTATGGTGTGTTCT	R- GGCAGGGGGCCTGGT
*17 (1023 C>T) rs28371706	F-TTCGGGGACGTGTTCAGC	F-CGCCTGTGCCCATCA
*17 (1023 C>T) rs28371706	R. Bio-CGGGTCCCACGGAAATCT	F-CGCCTGTGCCCATCA
*41 (4180 C>T) rs1135840	F-GAACCCTGAGAGCAGCTTCAAT	F-CCCCGCCTGTACCCT
*41 (4180 C>T) rs1135840	R.Bio-TATGTTGGAGGAGGTCAGGCTTAC	F-CCCCGCCTGTACCCT

### *CYP2D6* genotype and phenotype determination

All DNA samples were genotyped for CYP2D6*2, *3, *4, *6, *10, *17 and *41 variant alleles by pyrosequencing [12, 14]. All the pyrosequencing reactions were carried out on Pyrosequencing PSQ HS 96 platform according to the manufacturer’s recommendations (Qiagen, Valencia, CA, USA). The PCR reaction was performed in a final volume of 12.5 μl that consisted of; 6.5 μl HotStarTaq Master Mix (Qiagen), 1 μl of 10 pmole forward PCR primer, 1 μl of 10 pmole reverse PCR primer, 2 μl of H2O, and 2 μl of (20 ng/ μl) genomic DNA. The PCR reactions included 45 cycles with the following conditions: 95°C for 15 minutes followed by 95°C for 30 seconds, 60°C for 30 seconds, 72°C for 1 minute, and a final extension at 72°C for 7 minutes. The PCR and sequencing primers and annealing temperature for pyrosequencing assays are shown in table 2. The CYP2D6 metabolizer phenotype was inferred from the genotype information based on the activity score system recommended by the Clinical Pharmacogenetics Implementation Consortium (CPIC) guidelines [4].

### *CYP2D6* copy number determination

The *CYP2D6* gene copy number for all DNA samples was first determined by TaqMan Copy Number Assay (Life Technologies, CA), and then by the new pyrosequencing allele quantification-based method. For the TaqMan method, RNase P assay (ID: 431683) served as the internal control for copy number analysis (Life Technologies, CA). The primers used in this method were selected to target a specific sequence on *CYP2D6* exon 9 (TaqMan Copy Number Assay ID: Hs00010001_cn), and intron 6 (TaqMan Copy Number Assay ID: Hs04502391_cn). The *CYP2D6* exon 9 copy number assay was used to quantify all non-CYP2D6*36 alleles and the *CYP2D6* intron 6 copy number assay was used to detect and quantify all alleles including the CYP2D6*36 allele [11, 13]. All the samples were run in quadruplicates along with 4 Corriel DNA samples [15] with known copy number used as positive controls carrying a *CYP2D6* deletion (1 gene copy), and 2, 3, and 4 *CYP2D6* gene copies. The TaqMan copy number assay was performed according to the manufacturer’s recommendations and published protocol [11, 13]. Relative quantification of *CYP2D6* gene copy number was performed by using CopyCaller Software (Life Technologies, CA) following the comparative ΔΔC_T_ method [6, 13, 16].

After genotyping the DNA samples for *CYP2D6* *2, *3, *4, *6, *10, *17, *41 by pyrosequencing on PSQ HS 96 platform, the allele quantification application of the pyrosequencing was used to analyze and quantify the *CYP2D6* gene copy number, and also to identify which allele carried the duplication or multiplication. The variations in *CYP2D6* gene copy number were assessed by measuring the percentage of each allelic base at the polymorphic site which defines each variant allele by using the allele quantification (QA) option in the Pyrosequencing analysis software. The number of samples to be genotyped by this method can vary from 1 to 96 sample(s) at a time based on the workload.

## Statistical Analysis

The chi-squared test with one degree of freedom was used to test the departure from Hardy-Weinberg equilibrium (HWE) for each *CYP2D6* variant allele in different race/ethnic group. Mean allelic ratios are represented with standard deviations (SD). Data (means of allelic ratios) for samples with 2, 3, and 4 gene copies were compared by Analysis of Variance test (ANOVA), and P<0.05 was considered statistically significant. Post hoc analysis using Tukey’s test was done to identify the copy number associated with a mean allelic ratio that was significantly different from the others. Pearson’s correlation test was also performed to determine whether there is a positive linear correlation between gene copy number and allelic ratio. All statistical analysis were performed with SAS software (version 9.3, NC, USA)

## Results

### PCR and Pyrosequencing assay optimization

Since the genotyping and allele quantification are performed simultaneously in this method, the PCR reactions and pyrosequencing assays for *CYP2D6* *2, *3, *4, *6, *10, *17, *41 were optimized to be robust, efficient and reproducible. The annealing temperature for all PCR reactions was adjusted to 60ºC, and the annealing temperature for pyrosequencing reaction was kept at 80ºC for 3 minutes for all the assays. Under these conditions the DNA samples with known [13, 15] and unknown *CYP2D6* copy numbers [12] (2, 3, and 4) were repeatedly genotyped (at least 4 times on different days) to assure the reproducibility of our assay.

### Assay verification

DNA samples from Coriell Cell Repository (http://ccr.coriell.org/) with known *CYP2D6* copy numbers (2, 3, and 4 copies) and genotypes (Table 1) were used to verify the gene copy number by the new pyrosequencing quantification-based method. These samples were genotyped by pyrosequencing for *CYP2D6* *2, *3, *4, *6, *10, *17, *41 alleles and copy number variations. The *CYP2D6* genotypes and copy number variations results from the current method and known Coriell samples [13] (Table 1) were in 100% concordance. There was also no difference between pyrosequencing method and TaqMan copy number assay in determining the *CYP2D6* copy number variations.

### *CYP2D6* genotyping results

The genotypes and phenotypes of *CYP2D6* *2, *3, *4, *6, *10, *17, *41 alleles for all 218 PEAR-II DNA samples are shown in Table 3 [12]. All the genotypes were in Hardy-Weinberg Equilibrium (HWE). *CYP2D6* *1/*2 (18.8%) and *CYP2D6* *1/*1 (13.76%) were the most prevalent genotypes and made up 32.5% of the samples. The EM (84.4%), and UM (3.7%) phenotypes were the most and least frequent phenotypes respectively based on the CYP2D6 activity score.

**Table 3:** Distribution of CYP2D6 genotypes and phenotypes from 218 PEAR-II clinical samples ^1^.

**Genotype**	**n**	**Activity score**	**Phenotype**	**Phenotype frequency**
*3/*4	2	0	PM	5.0% (11)
*4/*4	7	0	PM	5.0% (11)
*4/*5	1	0	PM	5.0% (11)
*4/*6	1	0	PM	5.0% (11)
*3/*17	1	0.5	IM	6.9% (15)
*4/*10	2	0.5	IM	6.9% (15)
*4/*17	1	0.5	IM	6.9% (15)
*4/*41	7	0.5	IM	6.9% (15)
*5/*10	1	0.5	IM	6.9% (15)
*5/*17	3	0.5	IM	6.9% (15)
*1/*1	30	2	EM	84.4% (184)
*1/*2	41	2	EM	84.4% (184)
*1/*3	5	1	EM	84.4% (184)
*1/*4	16	1	EM	84.4% (184)
*1/*5	7	1	EM	84.4% (184)
*1/*6	2	1	EM	84.4% (184)
*1/*10	6	1.5	EM	84.4% (184)
*1/*17	6	1.5	EM	84.4% (184)
*1/*41	8	1.5	EM	84.4% (184)
*1×2/*4	2	2	EM	84.4% (184)
*1/*10×2	1	2	EM	84.4% (184)
*1/*17x2	1	2	EM	84.4% (184)
*2/*2	11	2	EM	84.4% (184)
*2/*4	18	1	EM	84.4% (184)
*2/*4×3	1	1	EM	84.4% (184)
*2/*5	6	1	EM	84.4% (184)
*2/*10	4	1.5	EM	84.4% (184)
*2/*17	5	1.5	EM	84.4% (184)
*2/*41	5	1.5	EM	84.4% (184)
*10/*17	2	1	EM	84.4% (184)
*10×2/*41	1	1.5	EM	84.4% (184)
*17/*17	3	1	EM	84.4% (184)
*17/*41	2	1	EM	84.4% (184)
*41/*41	1	1	EM	84.4% (184)
*1/*1×2	2	3	UM	3.7% (8)
*1x2/*2	2	3	UM	3.7% (8)
*1/*2×2	2	3	UM	3.7% (8)
*2×2/*10	1	2.5	UM	3.7% (8)
*2×2/*2	1	3	UM	3.7% (8)

^1^ These data were derived based on pyrosequencing and TaqMan copy number assay [13].

**Fig 1:**
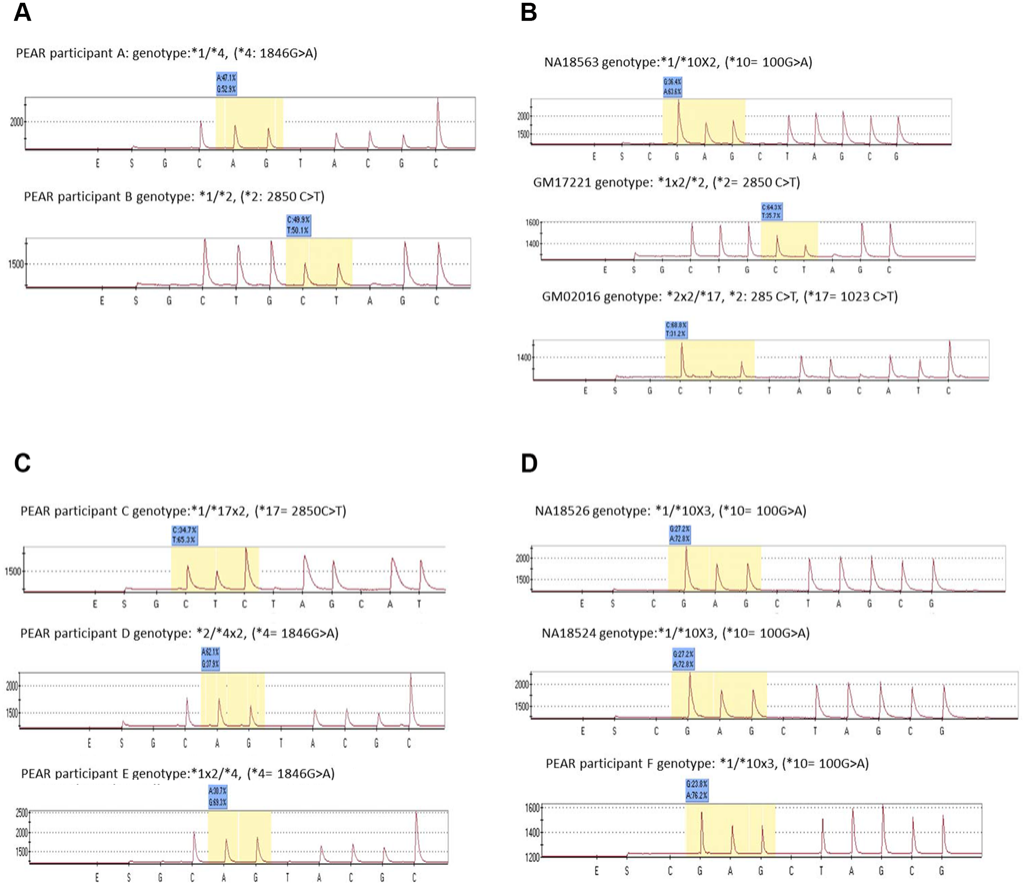
The pyrogram along with genotype calls and CNV (duplication/multiplication) and allele quantification (percent) for known and unknown Corriel and clinical DNA samples are shown in Fig. 1A, 1B, 1C, and 1D. **A.** Normal *CYP2D6* gene (no duplication). B. Coriell Samples with 3 known *CYP2D6* gene copies. The duplicated allele percentage ranges from 62% to 70%. **C.** Clinical samples with different *CYP2D6* gene copies. The duplicated allele percentage range from 62% to 70%. Without defining the duplicated allele, the predicted phenotypes for these individuals are ambiguous. **D.** Corriel and Clinical samples with 4 *CYP2D6* gene copies. The alleles with the multiplication (3X) show a percentage ranging from 72% to 76%.

The percent distribution of each allele specific nucleotide varied in a manner proportional with the copy number of its allele specifically in heterozygous cases with no gene copy number variations (Fig. 1A), the percent distribution of both alleles was equal or close to 50% whereas those with a total of 3 and 4 gene copy numbers, the pyrosequencing software yielded allele percent distributions of (62% to 70%), and (72% to 76%), respectively (Fig. 1B and 1D). There was no discordance between the results from our method (allele quantification-based Pyrosequencing), those from TaqMan copy number assay and reported known *CYP2D6* copy number samples.

Deriving accurate phenotypes from the genotype information by standard approaches that do not identify the duplicated/multiplicated allele (e.g. TaqMan copy number assay) was a challenge for 12 samples that had heterozygous genotypes and copy number variations in the *CYP2D6* gene. The inferred metabolic phenotype for each of the 12 samples varied depending on which of the two alleles existed in multiplications or duplications (Table 3). For example, one sample had a genotype of *1/*17 and a total gene copy number of 3. According to the recent CPIC guidelines for codeine therapy, the *17 variant allele is partially active and thereby is assigned an activity score of 0.5 whereas the wild type allele, *1, is given an activity score of 1 indicating a fully functional allele. Therefore, if the *1 allele is duplicated, then the predicted phenotype would be ultrarapid metabolizer (activity score = 2.5, greater than 2, the cut off for EM phenotype), whereas if the *17 allele is duplicated it would have an activity score = 2, thus an EM. Based on our method, the percent distribution of the two alleles (C and T) at the polymorphic position in this example were 65.3% and 34.7%, respectively, suggesting that the variant allele, *17 had a higher percent distribution or copy number in comparison with the wild type allele, *1 (Fig. 1C). Hence the genotype derived phenotype for this sample was EM and not UM (Table 4).

**Table 4:** *CYP2D6* genotypes, activity score and predicted phenotypes for samples included in pyrosequencing assay analysis.

**Genotype**	**Frequency (number of samples)**	**Predicted phenotype before pyrosequencing**	**Activity score**	**Predicted phenotype after pyrosequencing**	**Activity score**
1copy:					
*1/*5	1	EM	1	EM	1
*2/*5	1	EM	1	EM	1
2 copies:					
*1/*2	3	EM	2	EM	2
*1/*4	1	EM	1	EM	1
*2/*4	1	EM	1	EM	1
*2/*10	2	EM	1.5	EM	1.5
3 copies:					
(*1/*1)x3	1	UM	3	UM	3
(*2/*2)x3	1	UM	3	UM	3
*1/*2x2	6	UM	3	UM	2
*1/*17x2	1	EM or UM	2 or 2.5	EM	2
*2/*4x2	1	EM	1	EM	1
*1x2/*4	1	EM	1	EM	1
*2x2/*17	1	EM or UM	2 or 2.5	UM	2.5
*1/*10x2	5	EM or UM	2 or 2.5	EM	2
*2/*10x2	2	EM or UM	2 or 2.5	EM	2
4 copies:					
*1/*10x3	3	UM	2.5 or 3.5	UM	2.5
(*10/*10)x4	6	EM	2	EM	2

In homozygous states where there are 2 copies of the same *CYP2D6* allele, the percent distribution of the allele was about 100%. This method yields the same percentage (100%) for allele quantification in cases with one gene copy (carriers of the CYP2D6 *5 variant allele), or in homozygous samples with 2, 3, and 4 *CYP2D6* gene copies. The main limitation of this method is the inability to detect *5 and homozygous samples carrying more than 2 copies of *CYP2D6* gene. Of the 218 samples, 55 (25.2%) were homozygous for the *CYP2D6* gene and 18 (8.3%) were carriers of the *5 variant allele. Therefore, inferring the metabolizer phenotypes from the assigned *CYP2D6* genotypes for these samples was not possible by this method.

### *CYP2D6* allelic ratio

**Fig 2:**
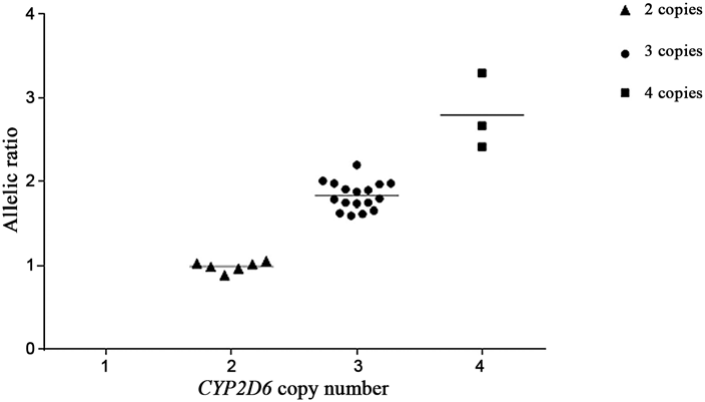
Scatter plot showing the mean allelic ratios for samples with 2, 3, and 4 *CYP2D6* gene copy numbers.

The mean allelic ratios for samples with 2, 3, and 4 gene copies were 0.97, 1.83, and 2.76, respectively (Table 5 and Fig. 2), and varied significantly with the number of the *CYP2D6* gene copy (ANOVA test, p = <0.0001). Additionally, Pearson’s correlation coefficient (r = 0.95) indicated a positive linear correlation between allele ratio and gene copy number.

**Table 5:** The mean allelic ratios for *CYP2D6* gene copy number.

**Gene copy number**	Mean allelic ratio *	**Standard deviation**	**Minimum**	**Maximum**
2 copies	0.97	0.07	0.85	1.06
3 copies	1.83	0.16	1.6	2.2
4 copies	2.76	0.39	2.42	3.2

* r = 0.95

## Discussion

In this study we describe a simple and reliable allele quantification-based Pyrosequencing genotyping method that facilitates *CYP2D6* CNV genotyping while also identifying allele-specific *CYP2D6* copy number variation in heterozygote samples. DNA samples carrying 2, 3, and 4 *CYP2D6* copy numbers were identified and also the allele which had the duplication or multiplication was determined. Determination of *CYP2D6* genotype as well as copy number is important in the clinical setting where phenotype prediction and individualization of drug therapy depends on the accuracy of the phenotypes that is inferred from genotype information.

In the current method for analyzing *CYP2D6* gene copy number, we used a different and novel strategy based on pyrosequencing. Our approach is simply based on calculating the ratio of the two *CYP2D6* alleles from the percentage (quantification) of each allele that is generated by the software at the end of the pyrosequencing reaction. Nevertheless, the means of the allelic ratios for samples with 2 and 3 gene copies were comparable to what was reported before (0.98 for 2 gene copies and 1.7 for three gene copies) [17]. Additionally, we were able to estimate the mean allelic ratio for samples that carried a total of 4 *CYP2D6* gene copies (allelic ratio = 2.7), which further underscores the importance of our approach in term of distinguishing between samples with 2, 3 and 4 copies of the *CYP2D6* gene as reported by others [6, 17].

TaqMan copy number assay is the most commonly used method for quantification of gene copy number mainly because of its simplicity in comparison with other complex assays such as long range PCR, RFLP, sequencing and Southern blotting [3, 6, 7, 8, 9, 13]. Its accuracy and reproducibility have been evaluated in several published studies [18]. Söderbäck et al, 2005, described a pyrosequencing-based method to determine *CYP2D6* gene copy number measuring the ratio of the *CYP2D6* and *CYP2D8* specific peak heights. They reported peak height ratios of 0.5 for samples with one copy of *CYP2D6* gene, 1 for samples with 2 copies and finally 1.5 for those with 3 gene copies.

Although there are different methods described in the literature for analyzing copy number variations [6, 17–20], they all share the same limitation, and that is their inability to determine which of the two *CYP2D6* alleles in a DNA sample carries duplication or multiplication, thereby adding more complexity to the process of inferring genotype derived phenotypes.

In contrast to long range PCR and sequencing or Southern-RFLP, which are labor intensive and time consuming, this method allows rapid estimation of gene copy number and most importantly identification of duplication/multiplication in heterozygous samples. One of the limitations of this assay is that, at present it cannot quantify gene copy number in samples that are homozygous for the *CYP2D6* gene or carry the *5 allele since only one *CYP2D6* allele is present for sequencing. The other limitation is that our assay cannot discriminate between CYP2D6*10 and CYP2D6*36 that share the same SNP (100 C>T). There is ongoing research in our lab to find a way to overcome these limitations.

To our knowledge, our method for determining copy number variations in *CYP2D6* has never been explored before. The method described here is straightforward, easy to perform, and reduces the cost as well as the time of genotyping significantly because it can identify the *CYP2D6* SNPs of interest and measure CNVs in a single reaction. Most importantly, it overcomes the limitations of the other techniques with regards to identifying the duplicated *CYP2D6* allele in heterozygous states, and thereby it allows faster prediction of the phenotypes from the genotypic data.
